# Appropriateness of Outpatient Antibiotic Use in Seniors across Two Canadian Provinces

**DOI:** 10.3390/antibiotics10121484

**Published:** 2021-12-03

**Authors:** Ariana Saatchi, Jennifer N. Reid, Marcus Povitz, Salimah Z. Shariff, Michael Silverman, Andrew M. Morris, Romina C. Reyes, David M. Patrick, Fawziah Marra

**Affiliations:** 1 Faculty of Pharmaceutical Sciences, University of British Columbia, Vancouver, BC V6T 1Z4, Canada; ariana.saatchi@ubc.ca; 2 Institute for Clinical Evaluative Sciences, London, ON N6A 5W9, Canada; jennifer.reid@ices.ca (J.N.R.); salimah.shariff@ices.on.ca (S.Z.S.); 3 Lawson Health Research Institute, London, ON N6C 2R5, Canada; michael.silverman@sjhc.london.on.ca; 4 Department of Medicine, University of Calgary, Calgary, AB T2N 1N4, Canada; marcuspovitz@gmail.com; 5 Faculty of Medicine, Division of Infectious Diseases, University of Western Ontario, London, ON N6A 3K7, Canada; 6 Department of Medicine, Sinai Health, University Health Network, University of Toronto, Toronto, ON N6A 3K7, Canada; andrew.morris@sinaihealthsystem.ca; 7 LifeLabs, Vancouver, BC V5Z 1H6, Canada; romina.reyes@lifelabs.com; 8 British Columbia Centre for Disease Control, Vancouver, BC V5Z 4R4, Canada; david.patrick@bccdc.ca; 9 School of Population and Public Health, University of British Columbia, Vancouver, BC V6T 1Z4, Canada

**Keywords:** antibiotics, antimicrobial drug resistance, outpatient care, inappropriate prescribing

## Abstract

Antimicrobials are among the most prescribed medications in Canada, with over 90% of antibiotics prescribed in outpatient settings. Seniors prescribed antimicrobials are particularly vulnerable to adverse drug events and antimicrobial resistance. The extent of inappropriate antibiotic prescribing in outpatient Canadian medical practice, and the potential long-term trends in this practice, are unknown. This study is the first in Canada to examine prescribing quality across two large-scale provincial healthcare systems to compare both quantity and quality of outpatient antibiotic use in seniors. Population-based analyses using administrative health databases were conducted in British Columbia (BC) and Ontario (ON), and all outpatient, oral antimicrobials dispensed to seniors (≥65 years) from 1 January 2000 to 31 December 2018 were identified. Antimicrobials were linked to an indication using a 3-tiered hierarchy. Tier 1 indications, which always require antibiotics, were given priority, followed by Tier 2 indications that sometimes require antibiotics, then Tier 3, which never require antibiotics. Prescription rates were calculated per 1000 population, and trends were examined overall, by drug class, and by patient demographics. Prescribing remained steady in both provinces, with 11,166,401 prescriptions dispensed overall in BC, and 27,656,014 overall in ON. BC prescribed at slightly elevated rates (range: 790 to 930 per 1000 residents), in comparison to ON (range: 745 to 785 per 1000 residents), throughout the study period. For both provinces, a Tier 3 diagnosis was the most common reason for antibiotic use, accounting for 50% of all indication-associated antibiotic prescribing. Although Tier 3 indications remained the most prescribed-for diagnoses throughout the study period, a declining trend over time is encouraging, with much room for improvement remaining. Elevated prescribing to seniors continues across Canadian outpatient settings, and prescribing quality is of high concern, with 50% of all antimicrobials prescribed inappropriately for common infections that do not require antimicrobials.

## 1. Introduction

The World Health Organization formally acknowledged the crisis of antimicrobial resistance (AMR) in 2014, and released a global action plan the next year [1]. In response, the United States, the United Kingdom (UK) and Australia have not only established jurisdiction-specific plans for the reduction of antibiotic use, but have also gone one step further and pledged to decrease inappropriate antibiotic use [2,3,4,5]. Despite the urgency, Canada has yet to disseminate a national action plan that focuses on the quality of antibiotic prescribing [6]. The continued knowledge gap on suboptimal prescribing quality precludes the scope of tangible targets for intervention, and research to parse inappropriate antibiotic use in Canada is vital [7,8].

Outpatient prescribing accounts for the majority of human antibiotic use in Canada, with 90% of all antibiotics dispensed in the community setting [8]. Evaluating appropriate antibiotic use is especially relevant for older Canadians (≥65 years), who are prescribed antimicrobials at elevated rates compared to other cohorts, particularly for respiratory tract infections (RTI) and urinary tract infections (UTI), with incidence of hospitalizations and “superbug” infections greater than in younger populations [9,10,11,12,13,14]. Historically, antibiotic use in seniors has been framed as a catch-22 situation, wherein both the prescription and/or reservation of antibiotics confer increased patient risk for adverse outcomes; however, recent studies counter this myth and report no association between reductions in antibiotic use and increased bacterial complications [15]. In the absence of documented patient harms, the stagnant rates of prescribing in Canadian seniors are especially alarming and require prompt attention to reduce antibiotic use within this population. In Canada, antibiotic receipt is the single most important risk factor for *Clostridioides difficile* infection and the acquisition of resistant infections, and it is the second most common cause of emergency department visits for adverse drug events [16,17]. Despite moderate declines in incidence across Canada since 2009, *C. difficile* remains one of the most burdensome pathogens, particularly in the elderly, causing more deaths than influenza each year [16].

On a global scale, the quantities of antibiotic use in humans, animals and agriculture have been surveilled for decades [18,19,20,21]. However, studies examining prescription quality have been hindered by the absence of encompassing guidelines, varying clinical factors and a high-level of expert subjectivity in defining inappropriate use. Moreover, the defining features of inappropriate use—antibiotic prescription in the absence of a bacterial etiology, the overuse of broad-spectrum agents, suboptimal dosing and/or duration of therapy—are diverse and difficult to aggregate [22]. Spivak et al. characterized early attempts to measure prescribing quality as trying to open a black box [22]. Since the publication of Spivak’s paper, several countries, including the United States and the UK have published criteria that define appropriateness; however, in Canada, inappropriate antibiotic use has remained largely unexamined and unknown. This study is the first in Canada to examine prescribing quality across two large-scale provincial healthcare systems to compare both quantity and quality of outpatient antibiotic prescription. Our objective was to evaluate trends of use over time in order to identify specific targets for subsequent intervention in a high-risk population.

## 2. Results

An average of 736,750 unique patients in Ontario (ON), and 587,705 in British Columbia (BC), were prescribed an antibiotic in any study year, with roughly 39 million total antibiotic prescriptions dispensed over the 19-year period (Table 1). The mean annual age of our cohort was 76 years in BC (SD: 8.3) and 75 years in ON (SD: 7.9). On average, about 38% of BC seniors were dispensed antibiotics in any study year, with 39% of ON seniors receiving prescriptions.

**Figure 1:**
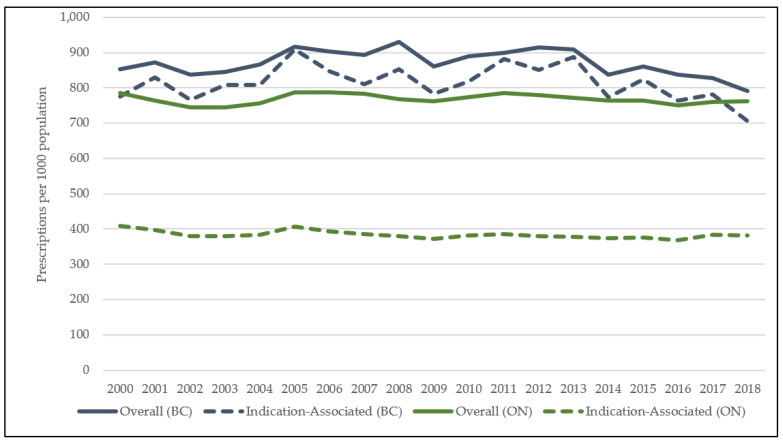
Rate of overall antibiotic use in British Columbia and Ontario, from 2000 to 2018.

As shown in Figure 1, oral antibiotics were prescribed at an overall, average rate of 768 prescriptions per 1000 population in ON, and 868 prescriptions per 1000 population in BC. Outpatient prescribing for ON seniors decreased by 2.8% over the study period (785 to 763 prescriptions per 1000 population), and by 7.5% in BC (854 to 790 prescriptions per 1000 population). In BC, 89% of all antibiotics dispensed were linked to common infections of interest, while in ON, 50% of antibiotics were associated with common infections in the outpatient setting. Indication-associated prescribing declined by a similar rate in both provinces over the study period (Supplement, Figure S1). In both ON and BC, prescribing for individuals aged ≥80 remained stable by 2018 with less than a 0.5% increase in both provinces (BC: 0.2%, ON: 0.4%), while prescribing for seniors aged 65–79 years declined (BC: 11%, ON: 5%). Additional data on prescribing by class and agent, by age and sex, is available in the online supplement (Supplement, Figures S2–S10).

### Primary Outcome: Indication-Associated Antibiotic Use & Inappropriate Prescribing

In both provinces, prescribing for Tier 1 diagnoses increased over the study period (BC: 44% (r = 0.96; *p* < 0.0001); ON: 28% (r = 1.00; *p* < 0.0001)) (Table 2). Urinary tract infections (UTI) accounted for most prescriptions within this tier, with 89 prescriptions issued per 1000 in ON, and 129 prescriptions per 1000 in BC, by 2018. Pneumonia-associated prescribing increased by roughly 10% in both provinces; however, this change was not significant in ON (BC: 11% (r = 0.93; *p* < 0.0001); ON: 7% (r = 0.90; *p* = 0.053)). Miscellaneous bacterial infections remained low in magnitude in ON (2018: 3 prescriptions per 1000 population); however, BC increased over the study period from 2 to 18 prescriptions per 1000 population by 2018 (r = 1.00; *p* < 0.0001).

Antibiotics were the least prescribed for Tier 2 diagnoses across both provinces (Table 2). By 2018, BC rates for this tier remained stable, while ON saw a 16% (r = 1.00; *p* < 0.0001) increase over the study period. Within the diagnoses that sometimes require antibiotics, skin and soft tissue infections (SSTI) and sinusitis were most prescribed. SSTI were prescribed at the highest rates throughout the study period: from 37 to 44 prescriptions per 1000 population in ON (r = 0.99; *p* < 0.0001) and 58 to 62 prescriptions per 1000 population in BC (r = 0.97; *p* < 0.0001). Other diagnoses within this tier included: pharyngitis, otitis media, acne, and gastrointestinal infections (GI), which were all prescribed at <5 prescriptions per 1000 population across both provinces.

In any study year, Tier 3 were the most linked diagnoses (Table 2). As these indications do not require antibiotics, as per clinical guidelines, all prescriptions are likely inappropriate. Tier 3 prescribing decreased by −25% (249 to 189 prescriptions per 1000 population (r = 0.88; *p* < 0.0001)) in ON, and −23% in BC (396 to 306 prescriptions per 1000 population (r = 0.89; *p* < 0.0001)). Across both provinces, inappropriate prescribing for respiratory infections led within this tier. However, while prescribing for bronchitis decreased by −33% (r = 0.52; *p* < 0.0001) in ON, a 3% (r = 0.95; *p* < 0.0001) increase was observed in BC by 2018. Viral upper respiratory tract infections (URTI) decreased across both provinces, by −38% (r = 0.036; *p* < 0.0001) and −56% (r = −0.80; *p* < 0.0001) in ON and BC, respectively. Other Tier 3 indications followed similar directional trends across provinces, with varying magnitudes, except for other genitourinary tract infections. This indication declined in ON by −8% (r = 0.99; *p* < 0.0001), with a 3% (r = 0.92; *p* < 0.0001) increase in BC.

The antibiotic classes prescribed for common infections varied over the study period; however, similarities were identified across provinces (Table 3). Decreasing trends were observed for the classes of sulfonamides and trimethoprim (ON: −46% (r = −0.28; *p* < 0.0001); BC: −37% (r = 0.97; *p* < 0.0001)), macrolides (ON: −30% (r = 0.48; *p* < 0.0001); BC: −44% (r = −0.28; *p* < 0.0001)), and quinolones (ON: −31% (r = 0.59; *p* < 0.0001); BC: −29% (r = 0.36; *p* < 0.0001)). The decrease in macrolides was related to a drop in erythromycin prescribing between 2000 and 2008 and a drop in clarithromycin use in the last decade. Beta-lactam penicillin (ON: 13% (r = 0.61; *p* < 0.0001); BC: 7% (r = 0.75; *p* < 0.0001)) use increased marginally in comparison to the more marked increase in the class of tetracyclines (ON: 45% (r = −0.48; *p* < 0.0001); BC: 103% (r = 0.91; *p* < 0.0001)) The only class that diverged in directional trend over the study period was other beta lactam antibiotics (i.e., cephalosporins), with a slight increase in ON (2% (r = 0.94; *p* < 0.0001)) but a decrease in BC (−13% (r = 0.75; *p* < 0.0001)). The largest increase for both provinces was identified in other antibacterials (ON: 239% (r = 1.00; *p* < 0.0001); BC: 109% (r = 0.97; *p* < 0.0001)), with nitrofurantoin being the primary antibiotic accounting for this.

## 3. Discussion

This is the first study in Canada to evaluate primary care antibiotic appropriateness across two large provinces. Across Ontario and BC, indications for which antibiotics are not required were the most heavily prescribed for seniors—In any study year, followed by antibiotic prescribing for Tier 1 where antibiotics are always required. Both provinces were similar in the agents prescribed for indications apart from tetracyclines—wherein BC prescribing eclipsed Ontario and overall prescribing for doxycycline in BC was six times more than in Ontario.

With respect to prescribing quality, Canadian seniors continue to be overprescribed antibiotics for conditions that do not warrant their use [14,23]. Despite the fact that antibiotic use is unnecessary, Tier 3 indications were prescribed at rates 2–4 times higher than other tiers. In both provinces, bronchitis received high levels of antibiotic prescription; however, while BC prescribing remained stable for this indication, Ontario saw a 30% reduction in antibiotic use. Although Tier 3 indications remained the most prescribed-for diagnoses throughout the study period, a declining trend over time is encouraging, with much room for improvement remaining. Robust and ongoing elevated prescribing for Tier 3 indications has been corroborated across multiple studies and geographic regions [11,24,25,26,27]. Despite the inefficacy of antibiotics for non-bacterial conditions, prescriptions issued for these diagnoses continue to dominate. In the US, 30% of outpatient prescribing is inappropriate; moreover, these rates have not significantly improved for adult populations since their initial reporting in 2016 [24,25]. In the United Kingdom, at least 20% of all antibiotics are used inappropriately in the outpatient setting [28,29]. Our study identified 49% of all indication-associated prescriptions in Ontario, and 53% in BC were dispensed inappropriately to Canadian seniors. A previous study examining a relatively small and select group of Canadian practitioners reported only 13% of prescriptions issued to seniors in Ontario were inappropriate [14]. The population wide sampling of our study provides a more accurate view of overall practices, and our methodology (i.e., diagnostic classification into Tiers 1, 2 and 3) aligns with previous published data from the United States and offers a more direct comparison with reported results, dissuading the notion that Canadian seniors receive less inappropriate or unnecessary prescriptions.

In comparing the most common agents used, Ontario has adopted increased use of penicillins over time, driven by the use of amoxicillin. This shift to the use of beta-lactams is accompanied by the decreased use of macrolides, erythromycin in the early days and, more recently, clarithromycin, quinolones, mostly related to ciprofloxacin, and sulfonamides/trimethoprim classes. Despite the aggregate stability in prescribing, the shifting landscape of common agents highlight trends of optimization, in the absence of reduced quantity. A decline in use for the same three classes is also reflected in BC; however, a complimentary increase has been observed in tetracycline antibiotics—prescribed at rates four times that of ON. One reason for this difference in magnitude may be doxycycline first entering the BC formulary in 2002, but only covered by the Ontario Drug Benefit Program (ODB) as of 2009 [30,31]. Additionally, we speculate that the suspected switch to the use of doxycycline for respiratory infections in BC may explain the interprovincial gap, where beta-lactams are the preferred agents in Ontario. Another reason for the disparity in tetracycline use might be the varying population demographics across provinces—with BC’s population older, on average [32]. Trends in antibiotic use for both provinces, across various patient demographics were comparable—with females aged 65–79 years, in urban settings and lower income quintiles being more likely to be prescribed (Supplement, Table S3). However, a difference in prescribing culture may also be culpable as Bugs and Drugs, the leading clinical practice guideline in BC, emphasizes tetracycline use more so than other comparable resources [33]. By contrast, Health Quality Ontario promotes the use of beta-lactam monotherapy, especially for cases of community-acquired pneumonia. The divergence in clinical guidelines and prescribing practice highlight a stark difference across two otherwise comparable healthcare systems. Provincial antibiograms and/or patient demographics might account for this difference in antibiotic use; however, this provincial variability is likely multifactorial, and not clearly elucidated.

Although optimization of drug choice, with narrowest spectrum and shortest duration being a central tenant of antimicrobial stewardship, this study highlights the reduction of unnecessary prescribing as an ongoing, high priority issue for Canadian seniors. Elevated antimicrobial use in Canadian seniors is especially concerning in the wake of prolonged antimicrobial stewardship (AMS) efforts. Formal evaluation of AMS impact was beyond the scope of this study; however, both provinces have endeavored to curb the unnecessary use of antibiotics. In BC, the Do Bugs Need Drugs campaign has been operating since 2005, offering accredited courses on appropriate antibiotic use, disseminating guidelines and physician resources [34]. By contrast, Public Health Ontario promotes the principles of AMS, put forth through federal initiatives and further adopted by the Choosing Wisely initiative in 2016 [35,36]. These initiatives promote conversations between clinicians and patients regarding unnecessary antibiotic use. Although some improvements in the trends of antibiotic classes used were identified in this study, there remains a high level of inappropriate prescribing in both provinces. In BC, AMS efforts have been attributed to an overall decline in antibiotic use; however, this trend is driven by younger populations with much work to be done in seniors [23].

This study has limitations inherent to all retrospective studies using administrative health data. Physician billing records were used to extract indication data to characterize antibiotic prescriptions. In BC, 89% of all prescriptions were linked to a physician record for a common infection, with only 50% of Ontario prescriptions linked. These rates were expected cross-provincially and demonstrate an improvement in linkage, in comparison to previous studies of Canadian outpatient care [37]. The unlinked prescriptions in Ontario could be evenly attributed to diagnoses beyond the scope of this study or no record. The latter are likely attributable to hospital and/or emergency department visits, with subsequent prescriptions dispensed in the community, not included within study scope. Although it is notable that Canadian primary-care physician claims data has a high positive-predictive value for diagnosis of common infections, including acute non-bacterial upper-respiratory infections (0.84, 95% CI: 0.81 to 0.88), differences in coding practices across provinces are not unknown [38]. In BC, the inflated use of certain Tier 3 codes (e.g., other-genitourinary-conditions) may signify an underlying systematic difference in provincial coding practices. Often utilized by prescribers for patient presentations that do not fulfill more specific diagnosis criterium (e.g., cystitis), elevated use of these codes raises alarms regarding prescribing or coding quality. These “other” diagnoses also exist for skin/soft tissue and respiratory tract infections. By 2018, prescribing for “other respiratory tract” infections halved in BC, although it was still twice the Ontario rate. The difference in scale for these indications, across otherwise comparable landscapes of primary care, compels further investigation into BC administrative data. Despite its routine utilization for research purposes, no previous studies validating BC administrative health data, for relevant ICD−9 codes, were identified by the authors. Furthermore, as the nature of our data prevents nested analyses, multiple prescriptions were permitted per individual, and our standard error may be biased. Rates do not account for unfilled prescriptions, and levels of compliance to medications are unknown. In the absence of lab data to confirm bacterial infection, or patient comorbidity data, our use of billing codes may be subject to misclassification bias. Finally, our study focused exclusively on patients aged >65. Substantial improvements in antibiotic prescribing in paediatrics have occurred, but assessments in other age groups are needed.

## 4. Materials and Methods

### 4.1. Data Sources

The BC Ministry of Health and ICES (formerly known as the Institute for Clinical Evaluative Sciences) in Ontario house several health care-related databases, which have comprehensive information on their populations. Antibiotic information was extracted from the BC PharmaNet, and Ontario Drug Benefit (ODB) program systems [30,39]. The Medical Service Plan (MSP) billing system and the Ontario Health Insurance Plan (OHIP) record all claims submitted by physicians for services provided to residents of BC and Ontario, respectively, including diagnostic codes [40]. BC data were extracted, anonymized, and made available to researchers by Population Data BC. Ontario datasets were linked using unique encoded identifiers and analyzed at ICES. These provincial databases have been used repeatably to evaluate antimicrobial use at the population-level [37,38].

### 4.2. Study Population

All residents of BC and ON aged ≥65 (<105) years, with a valid provincial health care number, from 1 January 2000 to 31 December 2018 were included. Antibiotic data, limited to oral use only (continuous <30-day supply) were matched to physician billing records using anonymized patient identifiers. Indication data were pulled from physician billing records in the outpatient setting [41,42]. Prescriptions were matched to a practitioner service date within a 5-day range. A three-tiered hierarchy was applied to link only the most relevant diagnostic code to the prescription (Supplement, Table S1) [24]. This hierarchy has been utilized in several studies, and it includes: Tier 1 indications (always require antibiotic prescription); Tier 2 (sometimes requiring); and Tier 3 (antibiotics unnecessary) [14,25,26,43]. If multiple codes were present within a given linkage period, then precedent was given to Tier 1. Prescriptions that did not match to any physician record were classified as unlinked, while those that matched to an indication beyond study scope were flagged as other diagnosis.

### 4.3. Outcomes & Statistical Analyses

Diagnostic codes were based on the ninth revision of the International Classification of Diseases developed by WHO, commonly referred to as ICD−9. Several codes were grouped within diagnostic categories and subsequent hierarchy tiers. Tier 1 includes: miscellaneous bacterial infections, pneumonia, and urinary tract infections. Tier 2 includes: acne, gastrointestinal infections, otitis media, pharyngitis, sinusitis, and skin and soft tissue infections. Tier 3 includes: asthma/allergy, bronchitis/bronchiolitis, influenza, miscellaneous bacterial infections, non-suppurative otitis media, other genitourinary conditions, other respiratory tract conditions, other skin, cutaneous and mucosal conditions, and viral upper respiratory tract infections. (Supplement, Table S1). Antibiotics were classified based on the Anatomical Therapeutic Chemical (ATC) classification system [44]. Seven major J01, antibiotic classes were included: tetracyclines (J01A); beta-lactams (J01C); other beta-lactams (J01D); sulfonamides and trimethoprim (J01E); macrolides, lincosamides and streptogramins (J01F); quinolones (J01M); and other antibacterials (J01X) (Supplement, Table S2).

Cohort demographics were first examined by age, sex and income quintiles as well as urban/rural status. Consumption rates were calculated as the number of prescriptions per 1000 population per year, using age- and gender-specific denominators for each province [32,45,46]. The primary study outcome was rate of prescribing for common infections, defined as: indication-associated antibiotic prescribing. Indication-associated prescribing was further stratified by a 3-tiered hierarchy to further characterize prescribing quality: Tier 1 (always required), Tier 2 (sometimes required) and Tier 3 (never required). Temporal trends in antibiotic use were evaluated using both the Spearman correlation coefficient and Poisson regression, where a two-sided *p*-value < 0.05 was considered significant. Baseline rates of antibiotic use were also examined for the cohort, by major ATC class, clinically relevant drugs and patient age and sex. All outcomes of interest with *n* < 6 were excluded from subsequent analyses to preserve subject anonymity. In both provinces, analyses were conducted using SAS version 9.4 (SAS Institute, Cary, NC, USA), with the addition of R version 4.0.2 in BC.

## 5. Conclusions

This is the first study in Canada to evaluate primary care antibiotic appropriateness across two large provinces. Across Ontario and British Columbia, indications for which antibiotics are not required were the most heavily prescribed for seniors—in any study year, followed by antibiotic prescribing for Tier 1 where antibiotics are always required. Although antibiotic use remains high, our study identified some positive changes in prescribing patterns for Canadian seniors, with optimizations in drug choice and reductions in prescribing for Tier 3 indications (i.e., never require antibiotics), over time. However, prescribing quality remains an issue of high concern, with approximately 50% of antibiotics, across two Canadian provinces, used inappropriately. Research to further delineate prescribing quality by dose, duration, and lab susceptibility is underway in both provinces.

## Figures and Tables

**Table 1:** Cohort Characteristics.

Cohort Characteristics (*N*)	British Columbia				Ontario			
	Overall	2000	2009	2018	Overall	2000	2009	2018
Total Unique Patients	4,858,511	201,610	244,228	319,057	13,998,138	601,759	703,900	946,499
Number of patients by age (%)								
65–79	3,389,791 (69.8%)	146,173 (72.5%)	167,001 (68.4%)	227,559 (71.3%)	9,980,422 (71.3%)	456,569 (75.9%)	494,359 (70.2%)	674,699 (71.3%)
80+	1,468,720 (30.2%)	55,437 (27.5%)	77,227 (31.6%)	91,498 (28.7%)	4,017,836 (28.7%)	145,190 (24.1%)	209,541 (29.8%)	271,800 (28.7%)
Number of patients by sex (%)								
Female	2,752,378 (56.7%)	114,265 (56.7%)	138,883 (56.9%)	178,936 (56.1%)	8,189,152 (58.5%)	355,282 (59.0%)	413,658 (58.8%)	545,627 (57.6%)
Male	2,103,436 (43.3%)	86,900 (43.1%)	105,257 (43.1%)	140,115 (43.9%)	5,809,106 (41.5%)	246,477 (41.0%)	290,242 (41.2%)	400,872 (42.4%)
Number of patients by income quintile ^1^ (%)								
Quintile ^2^ 1 (Lowest)	1,031,509 (21.2%)	47,707 (23.7%)	52,032 (21.3%)	64,684 (20.3%)	2,813,645 (20.1%)	128,790 (21.4%)	135,953 (19.3%)	192,716 (20.4%)
Quintile 2	979,783 (20.2%)	39,759 (19.7%)	50,011 (20.5%)	63,912 (20.0%)	2,938,637 (21.0%)	133,887 (22.2%)	144,402 (20.5%)	198,441 (21.0%)
Quintile 3	920,488 (19.0%)	36,471 (18.1%)	46,374 (19%)	61,574 (19.3%)	2,772,633 (19.8%)	120,285 (20.0%)	138,695 (19.7%)	188,395 (19.9%)
Quintile 4	888,518 (18.3%)	33,419 (16.6%)	44,544 (18.2%)	61,254 (19.2%)	2,670,403 (19.1%)	105,682 (17.6%)	140,380 (19.9%)	177,228 (18.7%)
Quintile 5 (Highest)	949,983 (19.6%)	36,246 (18.0%)	47,952 (19.6%)	63,341 (19.9%)	2,761,308 (19.7%)	111,744 (18.6%)	141,974 (20.2%)	187,176 (19.8%)
Missing ^3^	71,014 (14.6%)	7475 (3.7%)	2458 (1.0%)	2764 (0.9%)	40,261 (0.003%)	1371 (0.2%)	2496 (0.4%)	2543 (0.3%)
Number of patients by rural/urban status ^4^ (%)								
Rural	803,136 (16.5%)	32,303 (16.0%)	40,565 (16.6%)	54,185 (17.0%)	1,806,438 (12.9%)	87,610 (14.6%)	91,868 (13.1%)	110,068 (11.6%)
Urban	3,882,869 (80.0%)	162,064 (80.4%)	194,937 (79.8%)	254,485 (79.8%)	1,2178,924 (87.0%)	513,854 (85.4%)	612,025 (86.9%)	834,225 (88.1%)
Missing	172,506 (35.5%)	7243 (3.6%)	8726 (35.7%)	10,387 (3.3%)	12,896 (0.01%)	295 (0.0%)	7 (0.0%)	2206 (0.2%)
Total antibiotic prescriptions (*N*)	11,166,401	450,517	557,931	721,191	27,656,014	1,197,646	1,393,583	1,865,267
Total indication-associated prescriptions (*N*)	10,444,129	409,280	508,372	645,183	13,793,763	622,917	682,192	934,958
Total unlinked ^5^ prescriptions (*N*)	722,272	41,237	49,559	76,008	13,862,251	574,729	711,391	930,309

^1^ Population Data BC determined neighborhood income quintile (i.e., household size-adjusted measure of household income) using a postal code-based algorithm standardized by Statistics Canada; ^2^ Income quintiles are a relative measure across provinces, lowest = 0–20% and highest = 81–100% income bracket; ^3^ Missing represents absent or not applicable patient demographic information; ^4^ Rural status represents local population of 1000 to 29,999, urban status represents local population ≥30,000; ^5^ Those dispensation records that did not link to physician record within ±5 day period; All data presented as whole number (proportion).

**Table 2:** Rates of antibiotic utilization per 100,000 seniors, by indication, in British Columbia and Ontario.

Diagnosis Category	British Columbia					Ontario				
	2000 ^1^	2018	Percent Change ^2^	Spearman Coefficient	*p* Value ^3^	2000	2018	Percent Change	Spearman Coefficient	*p* Value
Tier 1	123	177	44	0.96	<0.0001	95	121	27	1.00	<0.0001
Miscellaneous bacterial	2	18	800	1.00	<0.0001	1	3	200	1.00	<0.0001
Pneumonia	27	30	11	0.93	<0.0001	27	29	7	0.9	0.053
Urinary tract infection	94	129	37	0.23	<0.0001	67	89	33	0.29	<0.0001
Tier 2	93	95	2	0.97	0.058	64	74	16	1.00	<0.0001
Pharyngitis	2	1	−50	−0.51	<0.0001	4	3	−25	0.95	0.76
Sinusitis	22	25	14	0.96	<0.0001	19	23	21	0.99	<0.0001
Otitis media	6	3	−50	−0.37	<0.0001	3	2	−33	0.60	<0.0001
Skin and soft tissue infection	58	62	7	0.97	<0.0001	37	44	19	0.99	<0.0001
Acne	1	1	0	0.26	<0.0001	1	1	0	0.97	<0.0001
Gastrointestinal infections	4	4	0	0.69	<0.0001	3	3	0	0.98	<0.0001
Tier 3	396	306	−23	0.89	<0.0001	249	187	−25	0.88	<0.0001
Asthma/allergy	17	9	−47	0.29	<0.0001	12	6	−50	−0.31	<0.0001
Bronchitis	79	81	3	0.95	<0.0001	95	64	−33	0.52	<0.0001
Influenza	5	3	−40	0.33	<0.0001	4	2	−50	−0.35	<0.0001
Non-suppurative OM	2	1	−50	−0.14	<0.0001	4	3	−25	0.72	<0.0001
Viral URTI	77	34	−56	−0.80	<0.0001	59	37	−37	0.36	<0.0001
Other respiratory tract	69	34	−51	−0.67	<0.0001	15	18	20	0.98	<0.0001
Other genitourinary conditions	87	90	3	0.92	<0.0001	25	23	−8	0.99	<0.0001
Other skin, cutaneous and mucosal conditions	60	53	−12	0.94	<0.0001	35	33	−6	0.96	<0.0001
Miscellaneous non-bacterial	1	2	100	0.92	<0.0001	1	1	0	0.99	<0.0001
Unlinked Antibiotics ^4^	78	83	6	0.38	<0.0001	377	380	1	1.00	<0.0001

^1^ Rates were calculated as prescriptions per 1000 population using relevant provincial denominators (BC Stats/Intellihealth ON); ^2^ Difference in rate of prescribing in 2018 when compared to 2000; ^3^ Refers to the testing for trends from 2000 to 2018; ^4^ Unlinked antibiotics refers to those prescriptions that did not match to a relevant physician billing record within ±5 days of dispensation; Abbreviations: OM—otitis media, URTI—upper respiratory tract; Tier I indications always require antibiotics, Tier 2 indications sometimes require antibiotics, Tier 3 rarely/never require antibiotics; Statistics reported included Spearman’s rank correlation coefficient and Poisson regression analyses; Double brackets identify non-significant results

**Table 3:** Utilization by major class for indication-associated antibiotics prescriptions in British Columbia and Ontario.

Antibiotic (ATC Class)	British Columbia					Ontario				
	2000 ^1^	2018	Percent Change ^2^	Spearman Coefficient	*p* Value ^3^	2000	2018	Percent Change	Spearman Coefficient	*p* Value
Overall (J01)	775	707	−9	0.95	<0.0001	408	382	−6	0.97	<0.0001
Tetracyclines (J01A)	33	67	103	0.91	<0.0001	5	7	40	−0.48	<0.0001
Beta-Lactam Penicillins (J01C)	129	138	7	0.75	<0.0001	82	93	13	0.61	<0.0001
Other Beta-Lactams (J01D)	153	134	−13	0.94	<0.0001	69	70	1	0.94	<0.0001
Sulfonamides & Trimethoprim (J01E)	71	45	−37	0.97	<0.0001	32	17	−47	−0.28	<0.0001
Macrolides, Lincosamides and Streptogramins (J01F)	148	82	−44	−0.28	<0.0001	105	74	−30	0.48	<0.0001
Quinolones (J01M)	192	136	−29	0.36	<0.0001	101	69	−32	0.59	<0.0001
Other Antibacterials (J01X)	50	105	109	0.97	<0.0001	15	52	247	1.00	<0.0001

^1^ Rates were calculated as prescriptions per 1000 population using relevant provincial denominators (BC Stats/Intellihealth ON); ^2^ Difference in rate of prescribing in 2018 when compared to 2000; ^3^ Refers to the testing for trends from 2000 to 2018; Abbreviations: ATC—anatomical therapeutic chemical, OM—otitis media, URTI—upper respiratory tract; Statistics reported included Spearman’s rank correlation coefficient and Poisson regression analyses.

## Data Availability

Restrictions apply to the availability of these data.

## Supplementary Materials

The following are available online at https://www.mdpi.com/article/10.3390/antibiotics10121484/s1, Table S1: diagnostic hierarchy, Table S2: antibiotics within anatomical therapeutic class, Table S3: cohort characteristics, Table S4: overall rate of antibiotic use by indication tier, and diagnosis, Table S5: rate of indication-associated antibiotic use by major ATC class, Figure S1: antibiotic prescriptions by age category, Figure S2: rate of overall antibiotic (J01) use by sex, Figure S3: antibiotic prescriptions by major ATC classification, Figure S4: tetracycline (J01A) antibiotic use, Figure S5: penicillin (J01C) antibiotic use, Figure S6: other beta-lactam (J01D) antibiotic use, Figure S7: sulfamethoxazole and trimethoprim (J01E) antibiotic use, Figure S8: macrolide, lincosamide and streptogramin (J01F) antibiotic use, Figure S9: quinolone (J01M) antibiotic use, Figure S10: other antibacterials (J01X) antibiotic use.
