# Supplementary material for: Appropriateness of Outpatient Antibiotic Use in Seniors across Two Canadian Provinces
Source: Antibiotics (Basel). 2021 Dec 3;10(12):1484. doi: 10.3390/antibiotics10121484 (PMC8698544; doi:10.3390/antibiotics10121484)

<u>Supplemental Tables & Figures</u>

Table S1. Diagnostic Hierarchy

TIER	Diagnosis Grouping
Tier I Diagnoses—*Always Requiring Antibiotic Prescription*	
1	Miscellaneous Bacterial Infections
1	Pneumonia
1	Urinary Tract Infections
Tier II Diagnoses—*Sometimes Requiring Antibiotic Prescription*	
2	Acne
2	Gastrointestinal Infections
2	Otitis Media
2	Pharyngitis
2	Sinusitis
2	Skin & Soft Tissue Infections
Tier III Diagnoses—*Never Requiring Antibiotic Prescription*	
3	Asthma, Allergy
3	Bronchitis, Bronchiolitis
3	Influenza
3	Miscellaneous Infections
3	Non-Suppurative Otitis Media
3	Other Genitouruniary Conditions
3	Other Respiratory Tract Conditions
3	Othe Skin, Cutaneous & Mucosal Conditions
3	Viral Upper Respiratory Tract Infection

Table S2. Antibiotics within Anatomical Therapeutic Class

J01A - TETRACYCLINES	J01F - MACROLIDES, LINCOSAMIDES AND STREPTOGRAMINS
J01AA02 - Doxycycline	J01FA09 - Clarithromycin
J01AA07 - Tetracycline	J01FA10 - Azithromycin
J01AA08 - Minocycline	J01FF01 - Clindamycin
J01C - BETA-LACTAM ANTIBACTERIALS, PENICILLINS	J01M - QUINOLONE ANTIBACTERIALS
J01CE02 - Phenoxymethylpenicillin	J01MA02 - Ciprofloxacin
J01CA04 - Amoxicillin	J01MA01 - Ofloxacin
J01CA01 – Ampicillin	J01MA12 – Levofloxacin
J01CF02 - Cloxacillin	J01MA06 – Norfloxacin
J01CR02 - Amoxicillin and enzyme inhibitor	J01MA14 - Moxifloxacin
J01D - OTHER BETA-LACTAM ANTIBACTERIALS	J01X - OTHER ANTIBACTERIALS
J01DB01 - Cephalexin	J01XE01 - Nitrofurantoin
J01DB05 - Cefadroxil	J01XX01 – Fosfomycin
J01DC02 - Cefuroxime	J01XE08 - Linezolid
J01DC04 - Cefaclor	A01A – Vancomycin (oral)
J01DD08 - Cefixime	J01XD01 - Metronidazole
J01E - SULFONAMIDES AND TRIMETHOPRIM	
J01EE01 - Sulfamethoxazole and trimethoprim	

Table S3. Cohort Characteristics

ON Cohort Characteristics	2000	2001	2002	2003	2004	2005	2006	2007	2008	2009	2010	2011	2012	2013	2014	2015	2016	2017	2018
Total Unique Patients	601,759	602,241	602,064	613,282	631,500	665,088	673,687	687,579	693,295	703,900	729,705	759,146	784,679	811,448	834,373	863,411	876,691	917,911	946,499
Number of patients by age																			
65-79	456,569	451,579	445,995	449,732	457,639	477,409	480,155	486,704	487,424	494,359	509,623	529,018	546,898	566,553	586,068	608,088	620,878	651,032	674,699
80+	145,190	150,662	156,069	163,550	173,861	187,679	193,532	200,875	205,871	209,541	220,082	230,128	237,781	244,895	248,305	255,323	255,813	266,879	271,800
Number of patients by sex																			
Female	355,282	356,373	356,012	362,268	373,251	391,947	396,506	404,271	407,396	413,658	427,818	442,896	457,954	472,782	485,010	501,656	507,520	530,925	545,627
Male	246,477	245,868	246,052	251,014	258,249	273,141	277,181	283,308	285,899	290,242	301,887	316,250	326,725	338,666	349,363	361,755	369,171	386,986	400,872
Number of patients by income quintile[1]																			
Quintile 1 (Lowest)	128,790	126,689	124,215	123,545	132,775	137,393	136,544	137,074	136,989	135,953	138,367	142,650	144,589	148,213	176,406	180,424	181,678	188,635	192,716
Quintile 2	133,887	133,220	131,288	132,689	134,009	140,164	140,827	142,658	143,125	144,402	148,809	153,282	157,633	161,695	178,824	184,448	185,380	193,856	198,441
Quintile 3	120,285	120,986	121,506	124,214	121,774	129,951	132,069	135,301	137,077	138,695	143,879	149,742	154,932	160,466	165,282	171,201	174,425	182,453	188,395
Quintile 4	105,682	107,160	109,131	113,592	119,297	126,406	129,640	133,612	135,755	140,380	146,607	154,283	161,417	167,947	151,184	158,193	161,831	171,058	177,228
Quintile 5 (Highest)	111,744	112,811	114,516	117,703	121,653	129,188	132,634	136,760	138,083	141,974	149,388	156,427	163,249	170,120	160,502	166,892	171,039	179,449	187,176
Missing[3]	1,371	1,375	1,408	1,539	1,992	1,986	1,973	2,174	2,266	2,496	2,655	2,762	2,859	3,007	2,175	2,253	2,338	2,460	2,543
Number of patients by rural/urban status[2]																			
Rural	32,303	32,775	32,798	34,413	35,644	37,046	37,985	39,410	39,410	41,138	40,565	42,157	43,876	47,179	48,933	49,971	48,933	49,971	51,363
Urban	162,064	164,730	164,261	169,053	175,648	183,996	187,563	191,777	191,777	200,745	194,937	203,266	208,489	222,988	229,574	240,236	229,574	240,236	247,554
Missing[3]	295	307	312	453	295	241	244	246	244	7	9	6	7	14	1,879	1,968	2,041	2,122	2,206
Total antibiotic prescriptions	1,197,646	1,185,824	1,177,492	1,199,410	1,238,567	1,313,663	1,341,356	1,362,020	1,369,453	1,393,583	1,453,495	1,511,956	1,560,435	1,608,743	1,646,274	1,701,304	1,724,726	1,804,800	1,865,267
Total indication-associated prescriptions	622,917	616,725	601,113	611,454	629,272	677,356	669,893	670,762	677,005	682,192	715,275	743,339	757,806	785,863	805,251	837,247	848,025	907,310	934,958
Total unlinked[4] prescriptions	574,729	569,099	576,379	587,956	609,295	636,307	671,463	691,258	692,448	711,391	738,220	768,617	802,629	822,880	841,023	864,057	876,701	897,490	930,309
BC Cohort Characteristics	2000	2001	2002	2003	2004	2005	2006	2007	2008	2009	2010	2011	2012	2013	2014	2015	2016	2017	2018
Total Unique Patients	201,610	205,038	204,526	211,154	219,248	229,284	234,064	240,015	251,097	244,228	254,650	261,926	280,273	287,501	288,586	300,818	309,533	315,903	319,057
Number of patients by age																			
65-79	146,173	146,753	144,014	146,995	151,699	158,241	160,808	164,955	164,955	171,854	167,001	174,159	179,968	193,402	201,457	210,984	201,457	210,984	219,130
80+	55,437	58,285	60,512	64,159	67,549	71,043	73,256	75,060	75,060	79,243	77,227	80,491	81,958	86,871	87,129	89,834	87,129	89,834	90,403
Number of patients by sex																			

Female	114,265	116,294	115983	120163	124807	130283	133,072	135,667	135667	142336	138883	144474	148624	158822	164,162	170,274	164162	170274	174701
Male	86,900	88,383	88210	90697	94219	98804	100,843	104,201	104201	108645	105257	110100	113233	121398	124,384	130,514	124384	130514	134819
Number of patients by income quintile[1]																			
Quintile 1 (Lowest)	47,707	48,244	47,124	48,097	48,868	50,246	51,050	51,303	51,303	53,198	52,032	53,659	54,088	57,044	58,689	60,660	58,689	60,660	62,505
Quintile 2	39,759	40,121	40,192	41,551	45,035	47,284	47,736	48,538	48,538	50,821	50,011	51,740	53,192	56,879	58,501	60,709	58,501	60,709	62,562
Quintile 3	36,471	37,192	36,979	38,391	41,971	43,846	44,962	45,781	45,781	47,873	46,374	47,839	49,507	53,212	55,197	57,992	55,197	57,992	59,489
Quintile 4	33,419	34,155	34,656	35,712	38,856	41,126	41,967	43,933	43,933	46,274	44,544	46,930	48,689	52,361	54,043	56,905	54,043	56,905	59,035
Quintile 5 (Highest)	36,246	36,889	37,519	38,984	41,082	43,216	44,779	46,625	46,625	48,985	47,952	50,989	52,877	57,083	58,208	60,749	58,208	60,749	62,328
Missing[3]	7,475	7,884	7,453	7,744	2,707	2,832	2,781	2,907	2,907	3,084	2,458	2,525	2,522	2,741	2,598	2,585	2,598	2,585	2,498
Number of patients by rural/urban status[2]																			
Rural	32,303	32,775	32,798	34,413	35,644	37,046	37,985	39,410	39,410	41,138	40,565	42,157	43,876	47,179	48,933	49,971	48,933	49,971	51,363
Urban	162,064	164,730	164,261	169,053	175,648	183,996	187,563	191,777	191,777	200,745	194,937	203,266	208,489	222,988	229,574	240,236	229,574	240,236	247,554
Missing[3]	7,243	7,533	7,467	7,688	7,956	8,242	8,516	8,828	8,828	9,214	8,726	9,227	9,561	10,106	10,079	10,611	10,079	10,611	10,616
Total antibiotic prescriptions	450,517	469,471	458,020	471,274	492,844	533,269	540,937	548,606	548,606	585,897	558,109	593,442	618,990	656,790	652,816	699,028	652,816	699,028	707,450
Total indication-associated prescriptions	409,280	445,746	419,742	451,753	460,850	528,382	507,548	497,752	497,752	537,065	508,372	545,644	606,538	610,460	603,214	670,038	603,214	670,038	645,271
Total unlinked[4] prescriptions	41,237	23,725	38,278	19,521	31,994	4,887	33,389	50,854	50,854	48,832	49,737	47,798	12,452	46,330	49,602	28,990	49,602	28,990	62,179

1 Neighbourhood income quintile (i.e. household size-adjusted measure of household income) calculated using a postal code-based algorithm standardized by Statistics Canada;
2 Rural status represents local population of 1000 to 29,999, urban status represents local population ≥30,000;
3 Missing represents absent or not applicable patient demographic information.
4 Those dispensation records that did not link to physician record within ±5 day period

Table S4. Overall rate of antibiotic use by indication tier, and diagnosis, in British Columbia & Ontario, from 2000 to 2018

	Province	Year	Tier[3] I Overall	Miscellaneous bacterial infections	Pneumonia	Urinary tract infections	Tier II Overall	Pharyngitis	Sinusitis	Otitis media	Skin & soft tissue infections	Acne	Gastrointestinal infections	Tier III Overall	Asthma/allergy	Bronchitis	Influenza	Non-suppurative OM	Viral URTI	Other respiratory tract infections	Other genitourinary conditions	Other skin, cutaneous and mucosal conditions	Miscellaneous non-bacterial infections	Unlinked Antibiotics[2]
Rate[1] of Antibiotic Use (prescriptions per 1000 population)	British Columbia	2000	123	2	27	94	93	2	22	6	58	1	4	396	17	79	5	2	77	69	87	60	1	78
		2001	134	2	27	105	100	3	25	6	61	1	4	417	16	83	3	2	80	73	96	62	1	44
		2002	132	2	28	102	99	2	23	6	63	1	4	372	15	74	3	2	68	63	93	54	1	70
		2003	148	3	31	114	103	3	23	5	68	1	4	390	15	76	3	2	73	66	102	54	1	35
		2004	159	3	31	125	101	3	24	5	64	1	4	386	14	74	2	2	69	62	108	53	1	56
		2005	178	3	36	139	113	3	27	6	70	1	6	436	15	89	3	2	79	70	117	60	2	60
		2006	180	4	34	143	110	2	25	5	71	1	5	392	13	79	2	2	65	58	113	58	2	56
		2007	173	4	32	137	105	2	25	5	67	1	5	372	12	78	2	1	61	53	106	57	2	83
		2008	192	4	35	152	110	2	26	5	70	1	6	386	13	82	2	2	62	54	112	56	2	77
		2009	185	5	31	149	101	2	24	4	64	1	5	344	12	74	4	1	49	44	105	53	1	77
		2010	195	7	31	157	107	2	26	4	68	1	5	357	13	83	2	1	49	44	110	53	2	72
		2011	216	11	41	165	115	2	30	5	72	1	5	383	13	91	2	1	53	48	117	56	2	18
		2012	217	12	36	169	109	2	26	4	71	1	5	361	11	86	2	1	48	44	112	56	2	65
		2013	228	16	39	173	112	2	28	4	72	1	5	377	12	92	2	1	49	46	116	56	2	22
		2014	205	15	33	157	96	2	25	4	62	1	4	325	11	79	2	1	42	40	100	49	2	64
		2015	215	18	37	160	104	2	27	4	66	1	4	348	11	87	3	1	44	41	104	54	2	36
		2016	200	18	34	148	99	1	27	3	63	1	4	321	11	81	2	1	40	39	94	52	2	74
		2017	196	17	36	143	102	1	27	3	65	1	4	337	10	93	3	1	39	37	96	55	2	45
		2018	177	18	30	129	95	1	25	3	62	1	4	306	9	81	3	1	34	34	90	53	2	83
	Ontario	2000	95	1	27	67	64	4	19	3	37	1	3	249	12	95	4	4	59	15	25	35	1	377
		2001	93	1	26	66	65	4	20	3	37	1	3	239	11	92	2	4	57	15	24	35	1	367
		2002	94	1	26	67	65	3	19	2	38	1	3	222	10	84	2	3	51	14	24	34	1	365
		2003	95	1	25	69	65	4	20	2	38	1	3	220	9	83	2	3	51	14	24	33	1	365
		2004	98	1	25	72	66	3	19	2	39	1	3	220	9	83	2	3	49	14	25	34	1	372
		2005	104	1	27	75	69	4	20	2	41	1	3	233	9	88	2	3	54	16	26	36	1	381
		2006	103	1	25	76	68	4	19	2	41	1	3	223	9	82	1	3	50	15	26	36	1	394
		2007	102	1	25	76	68	4	20	2	40	1	4	215	8	79	1	3	48	14	25	35	1	397
		2008	101	1	24	76	66	3	20	2	39	1	4	212	8	78	1	3	48	15	25	34	1	388
		2009	102	1	23	78	66	3	20	2	40	1	3	204	7	73	2	3	45	15	25	34	1	389
		2010	107	2	25	80	69	3	20	2	41	1	4	205	7	73	1	3	44	15	25	35	1	393
		2011	108	2	26	80	70	4	21	2	42	1	4	207	7	74	1	3	45	16	24	36	1	399
		2012	109	2	26	81	70	3	20	2	42	1	4	200	7	71	1	3	43	16	24	35	1	401
		2013	112	2	26	83	70	3	21	2	43	1	4	195	7	70	1	3	41	16	23	32	1	395
		2014	113	3	26	84	70	3	21	2	42	1	3	190	7	67	1	3	41	16	23	32	1	390

		2015	116	3	28	85	71	3	21	2	42	1	3	189	7	67	1	3	39	16	23	32	1	388
		2016	116	3	26	87	72	4	22	2	43	1	3	181	6	62	1	3	37	16	23	32	1	381
		2017	119	3	28	88	74	4	24	2	43	1	3	189	7	67	1	3	39	17	22	32	1	378
		2018	121	3	29	89	74	3	23	2	44	1	3	187	6	64	2	3	37	18	23	33	1	380

1Rates were calculated as prescriptions per 1000 population using relevant provincial denominators (BC Stats/Intellihealth ON); 2 Unlinked antibiotics refers to those prescriptions that did not match to a relevant physician billing record within ±5 days of dispensation; 3 Tier 1 indications always warrant antibiotic use, Tier 2 indications sometimes warrant antibiotic use, Tier 3 indications never warrant antibiotic use; Abbreviations: URTI-upper respiratory tract infections, OM-otitis media;

Table S5. Rate of indication-associated antibiotic use by major ATC class in BC & ON, from 2000 to 2018

	Province	Year	Major Antibiotic Class (ATC Code)							
			Overall (J01)	Tetracyclines (J01A)	Beta-Lactam Penicillins (J01C)	Other Beta-Lactams (J01D)	Sulfonamides & Trimethoprim (J01E)	Macrolides, Lincosamides, Streptogramins (J01F)	Quinolones (J01M)	Other Antibacterials (J01X)
Rate[1] of Antibiotic Use (prescriptions per 1000 population)	British Columbia	2000	775	33	129	153	71	148	192	50
		2001	829	32	132	147	67	170	223	57
		2002	767	27	115	131	55	162	224	52
		2003	809	26	117	136	55	168	245	63
		2004	809	24	112	134	53	159	253	74
		2005	909	28	124	150	57	182	280	89
		2006	847	27	113	142	56	159	260	90
		2007	811	27	108	133	54	155	244	90
		2008	852	31	118	136	62	157	249	99
		2009	784	30	102	127	57	131	239	97
		2010	817	38	117	121	60	134	238	110
		2011	881	44	123	142	58	141	244	128
		2012	850	43	120	142	54	129	227	133
		2013	887	51	138	146	57	129	226	139
		2014	773	50	123	128	51	105	194	123
		2015	824	59	138	138	51	110	196	132
		2016	763	59	130	134	48	99	170	124
		2017	782	68	142	143	47	98	156	129
		2018	707	67	138	134	45	82	136	105
	Ontario	2000	408	5	82	69	32	105	101	15
		2001	397	4	81	67	34	112	80	20
		2002	380	3	72	63	31	107	82	21
		2003	380	3	70	62	27	107	88	23
		2004	384	3	68	64	26	105	93	26
		2005	406	2	71	68	26	110	100	28
		2006	393	2	69	65	23	100	105	29
		2007	385	2	67	64	22	97	104	30
		2008	380	2	63	62	21	98	103	31
		2009	373	2	63	62	21	92	100	33
		2010	381	1	68	58	22	94	103	35
		2011	386	1	68	64	21	93	101	37
		2012	379	1	69	65	19	91	96	38
		2013	377	1	72	67	20	85	93	39
		2014	373	1	76	67	21	80	88	40
		2015	376	1	81	68	19	79	86	43
		2016	369	1	83	69	18	74	76	47
		2017	382	2	93	71	18	77	71	50
		2018	382	7	93	70	17	74	69	52

[1] Rates were calculated as prescriptions per 1000 population using relevant provincial denominators (BC Stats/Intellihealth ON)

Abbreviations: ATC-Anatomical Therapeutic Classification

Figure S1. Rates of antibiotic prescriptions by age category, in BC & ON, from 2000 to 2018

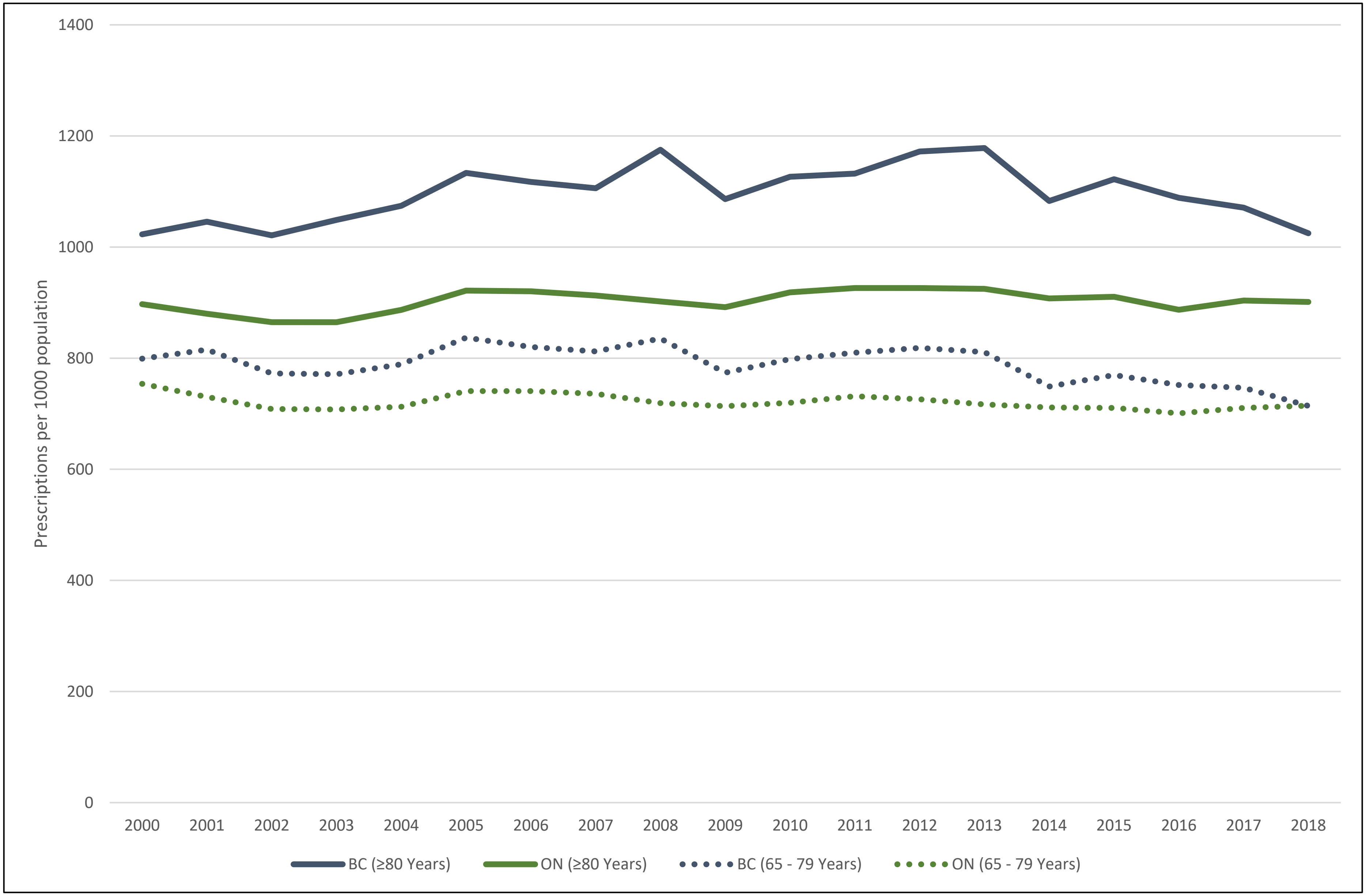

Figure S2. Rate of overall (J01) antibiotic use in British Columbia & Ontario, by sex, from 2000 to 2018

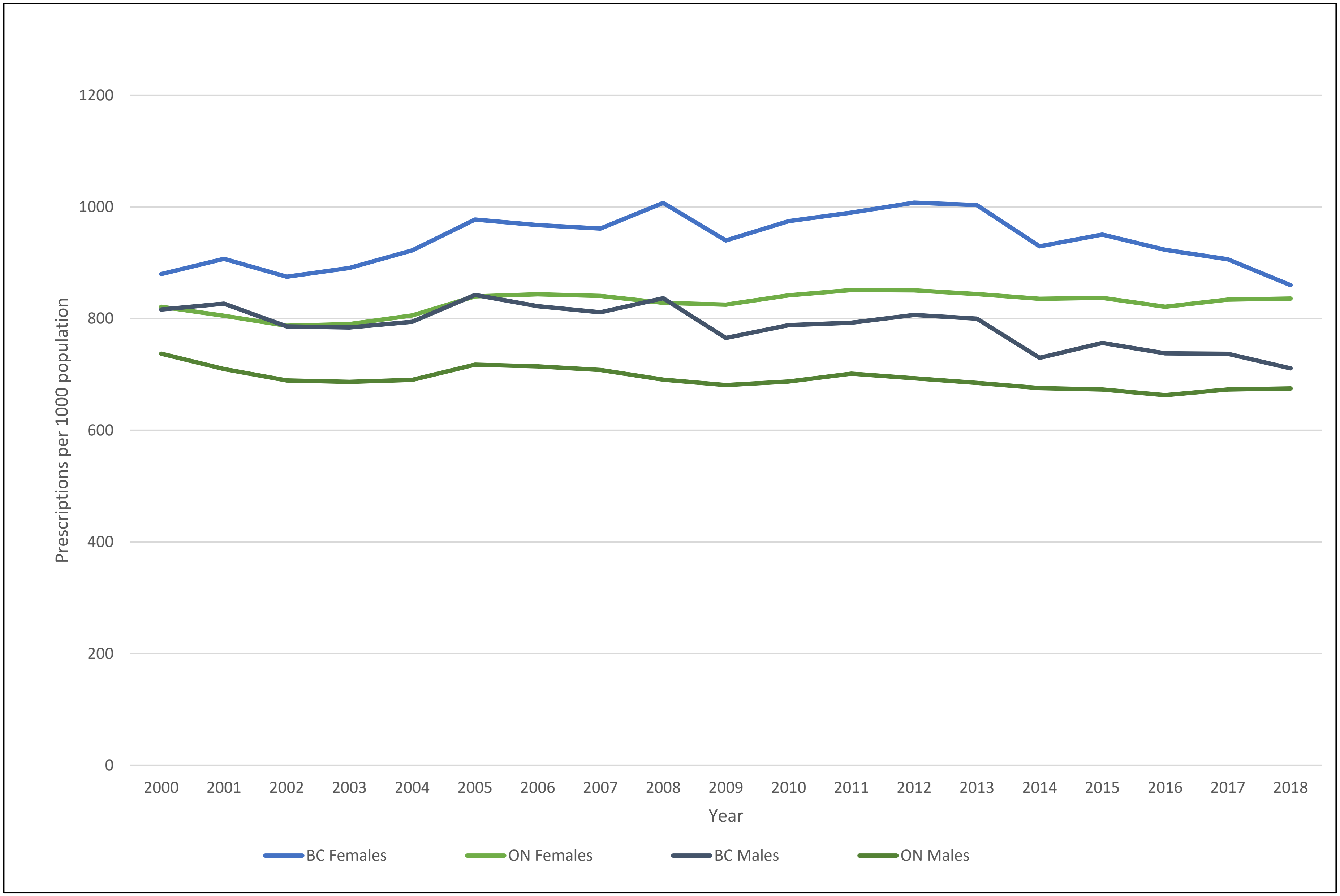

Figure S3. Antibiotic prescriptions by major ATC classification in BC & ON, from 2000 to 2018

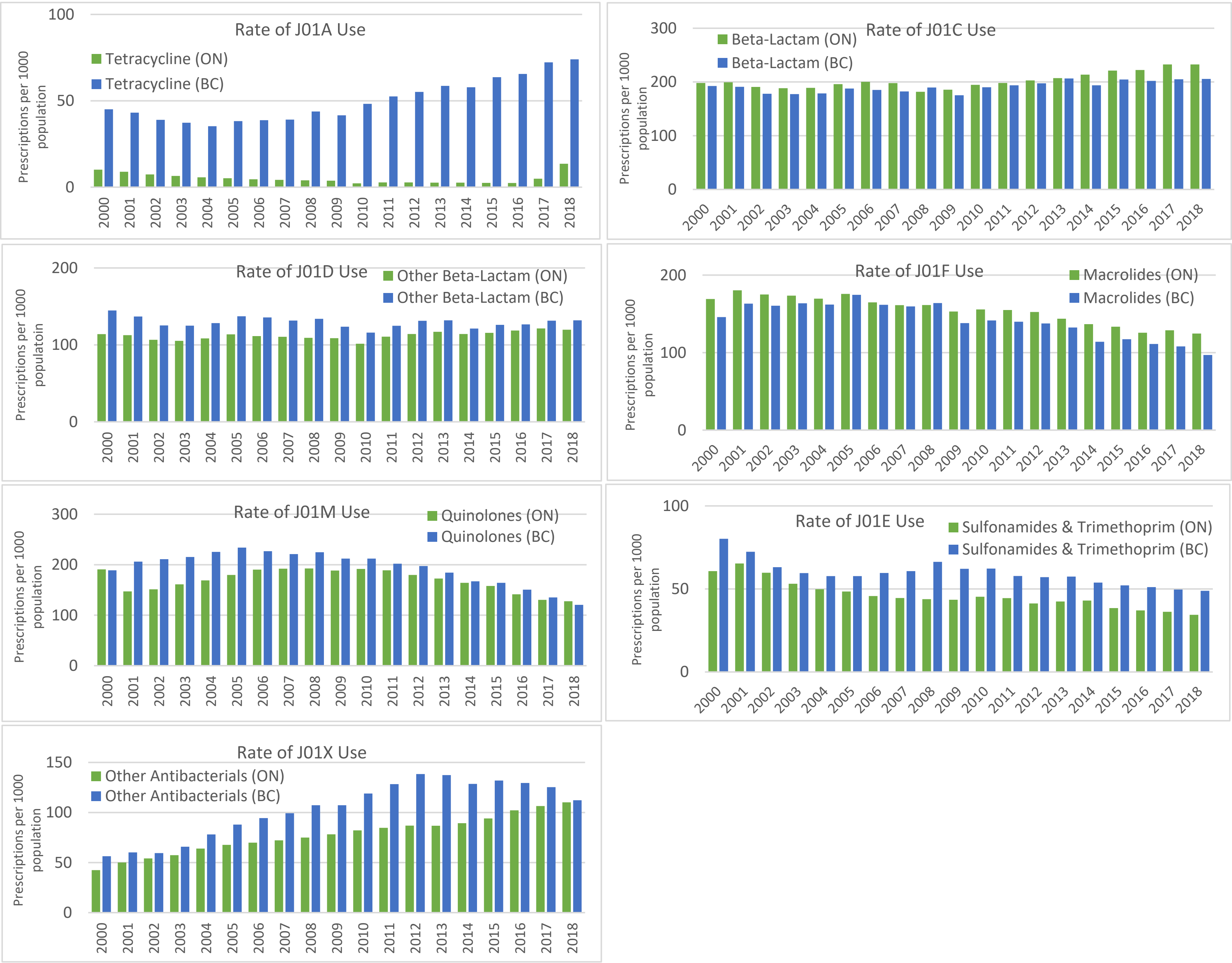

Figure S4. Tetracycline (J01A) antibiotic use in Ontario & British Columbia from 2000 to 2018

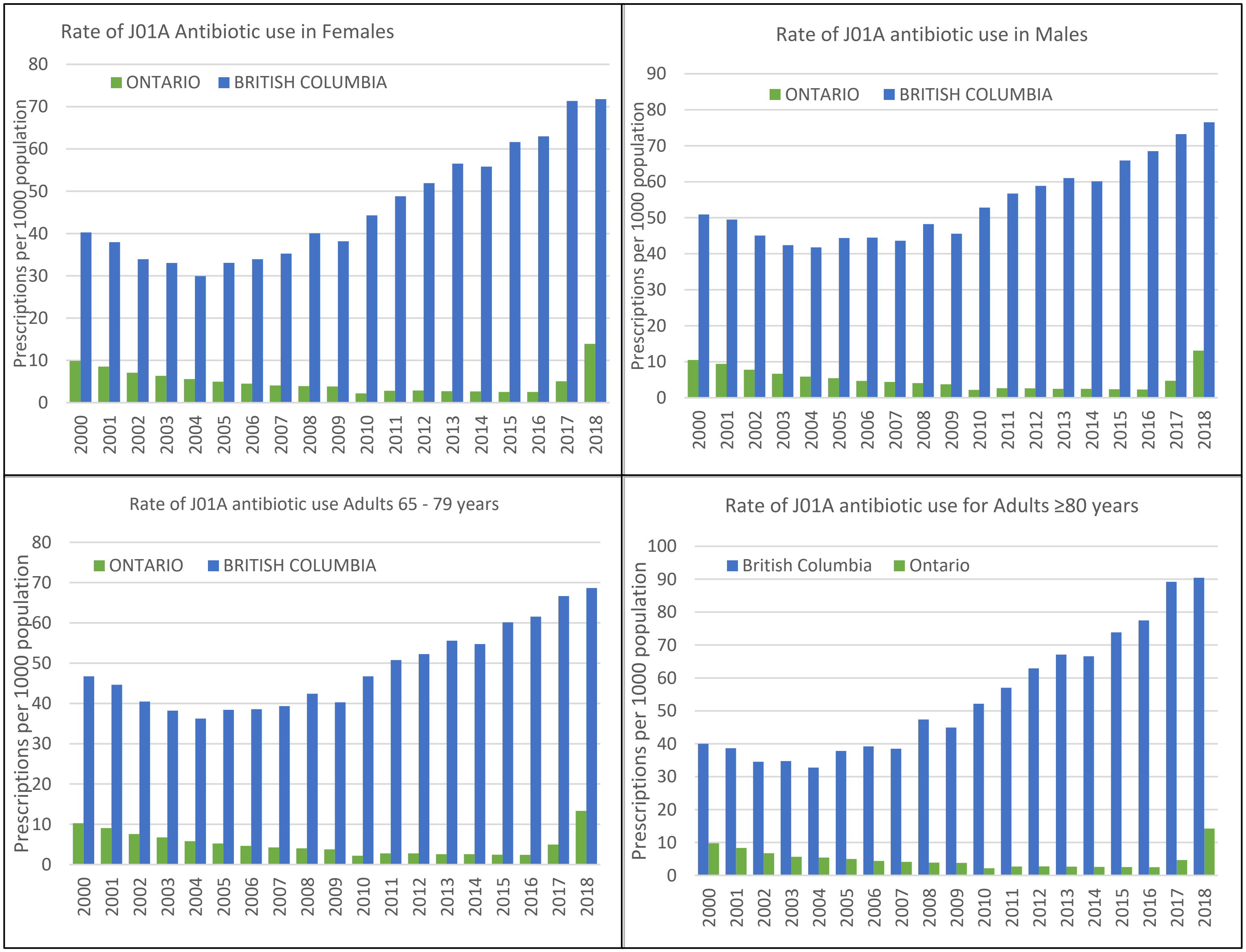

Figure S5. Penicillin (J01C) antibiotic use in Ontario & British Columbia from 2000 to 2018

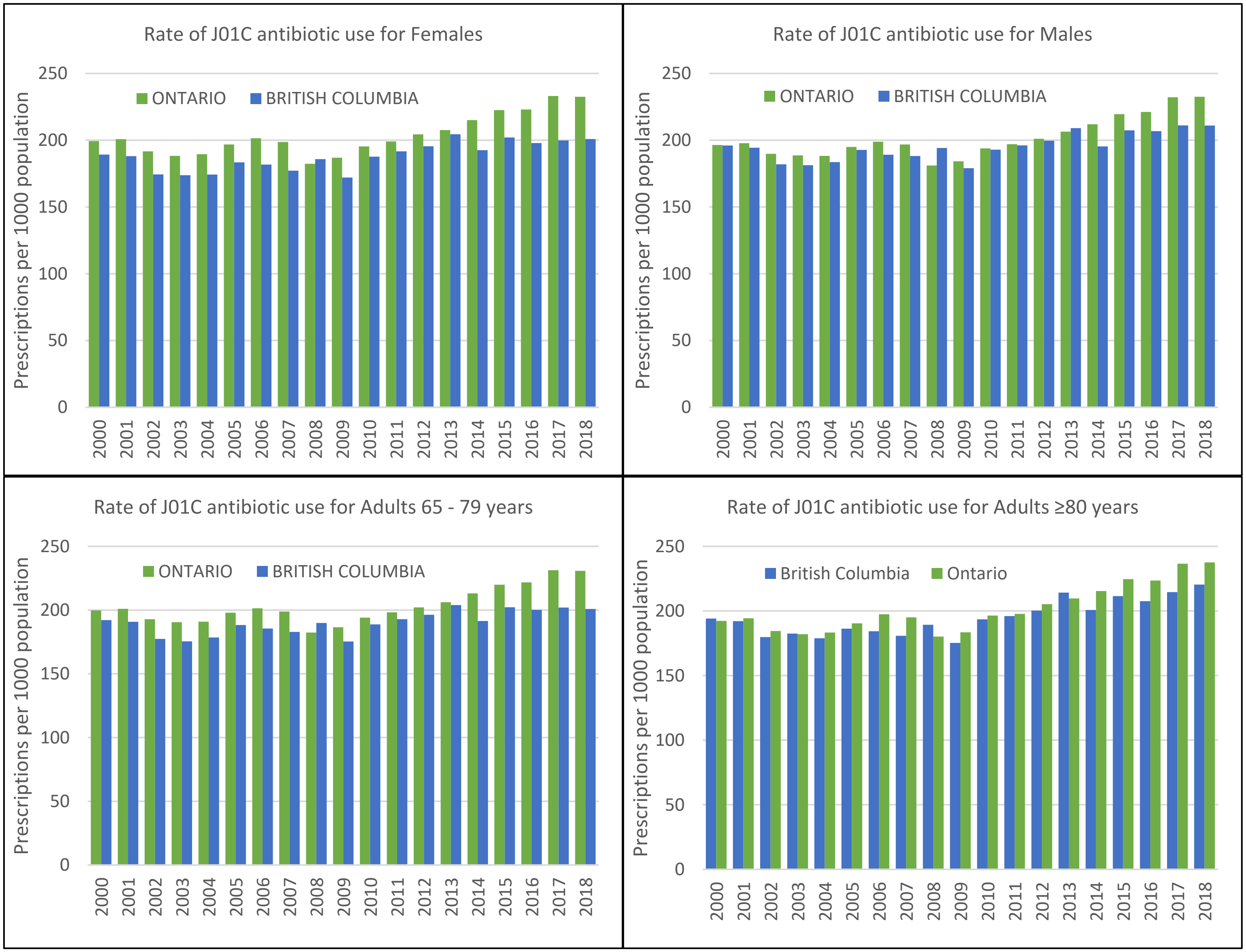

Figure S6. Other Beta-Lactam (J01D) antibiotic use in Ontario & British Columbia from 2000 to 2018

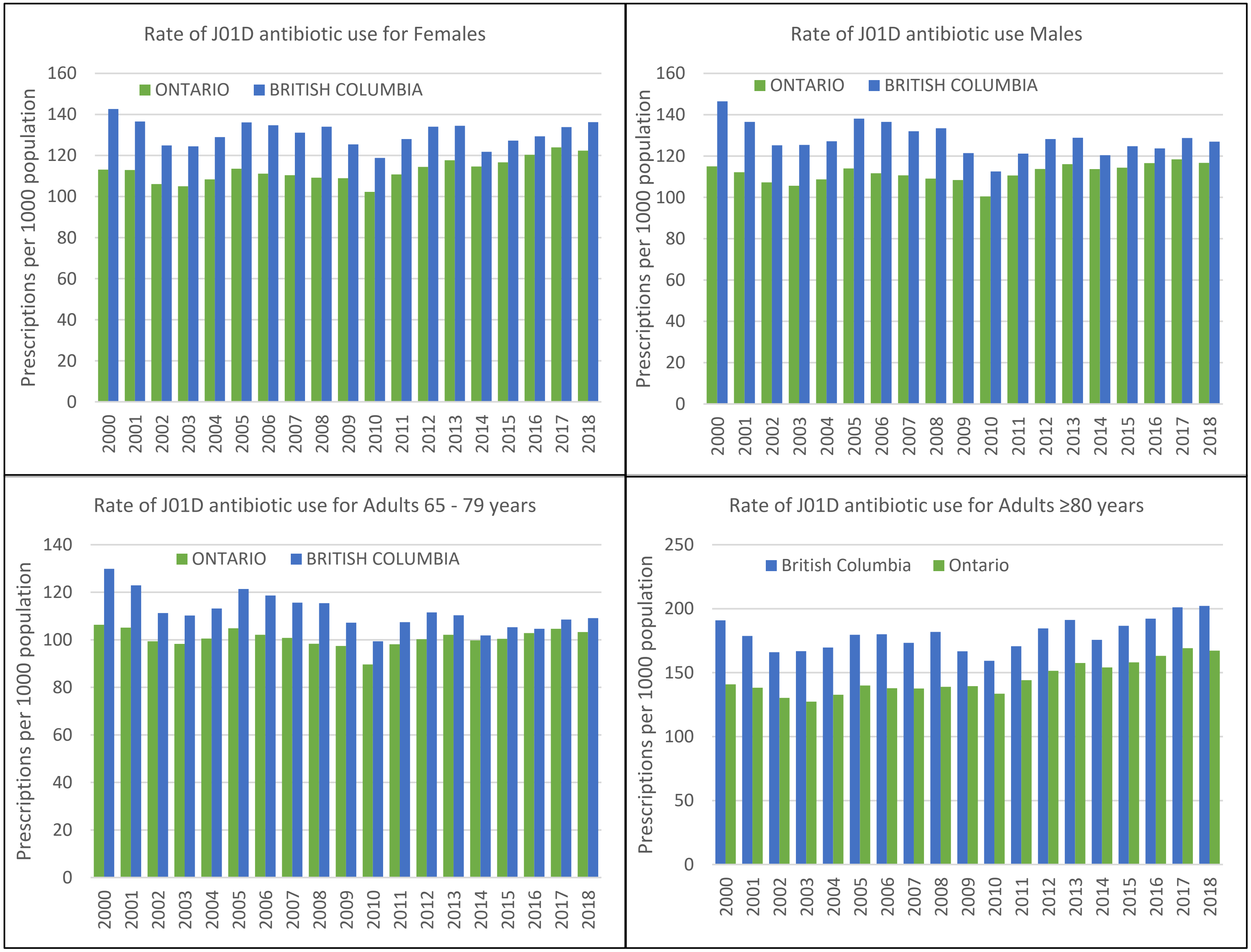

Figure S7. Sulfamethoxazole & Trimethoprim (J01E) antibiotic use in Ontario & British Columbia from 2000 to 2018

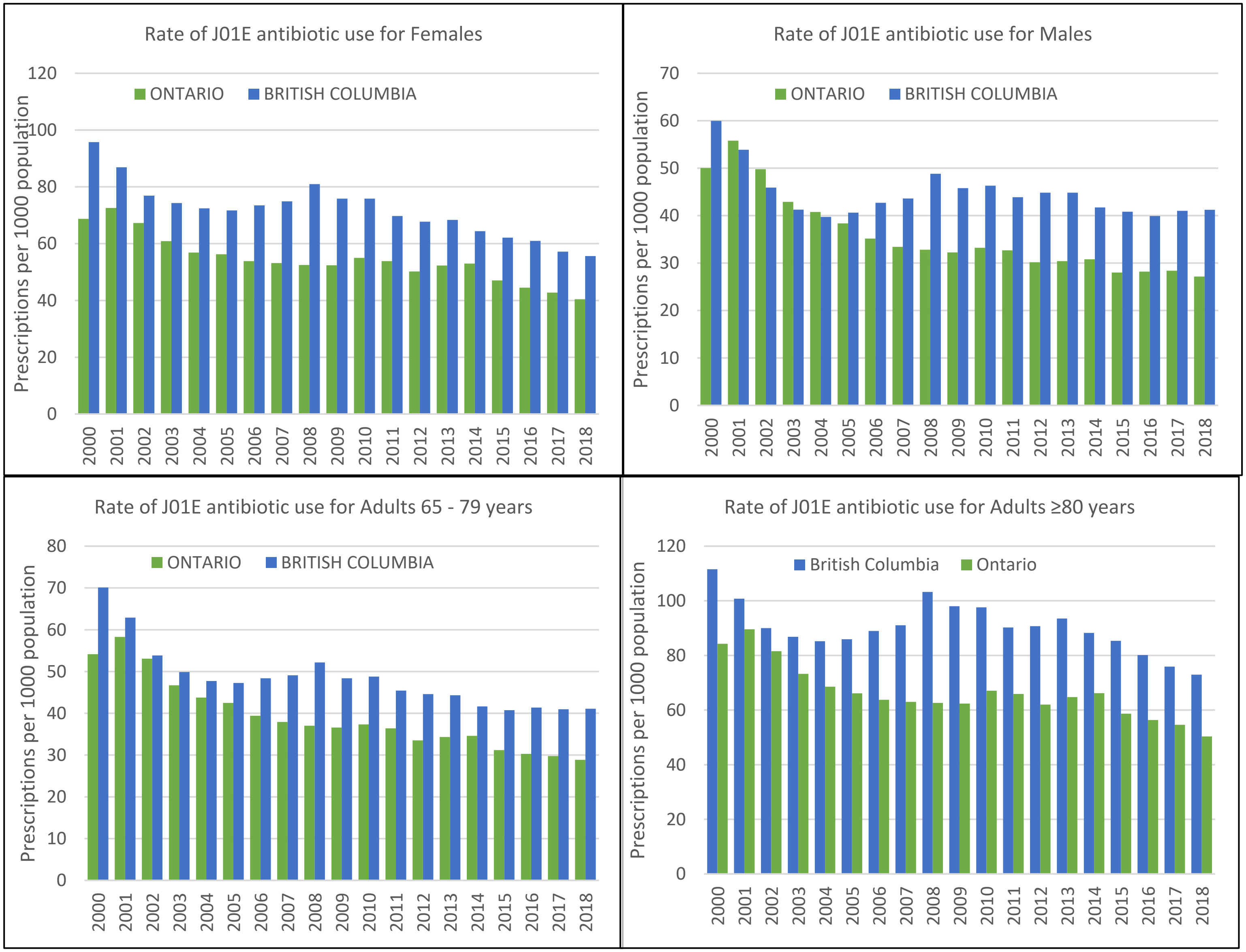

Figure S8. Macrolide, Lincosamide & Streptogramin (J01F) antibiotic use in Ontario & British Columbia from 2000 to 2018

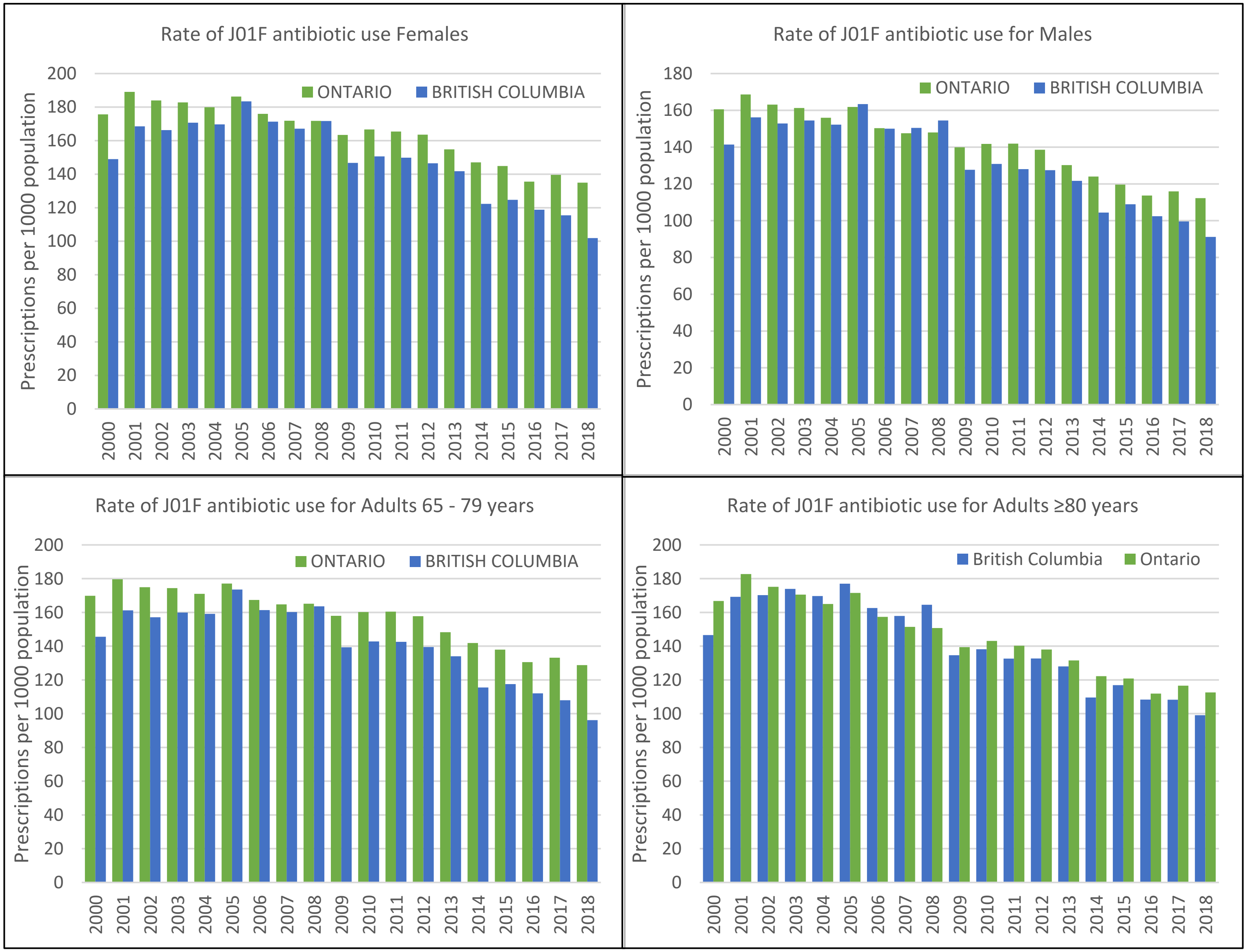

Figure S9. Quinolone (J01M) antibiotic use in Ontario & British Columbia from 2000 to 2018

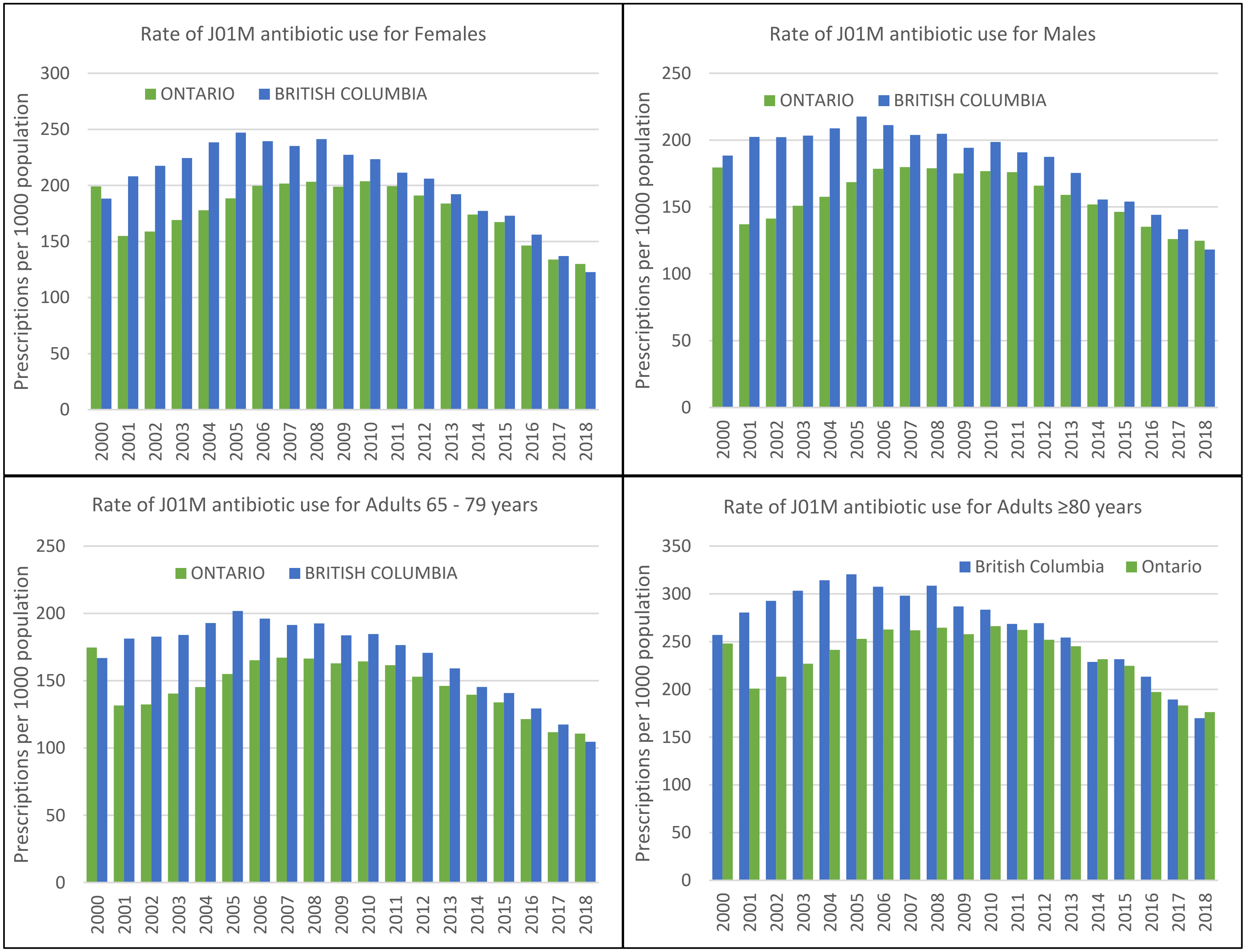

Figure S10. Other Antibacterials (J01X) antibiotic use in Ontario & British Columbia from 2000 to 2018

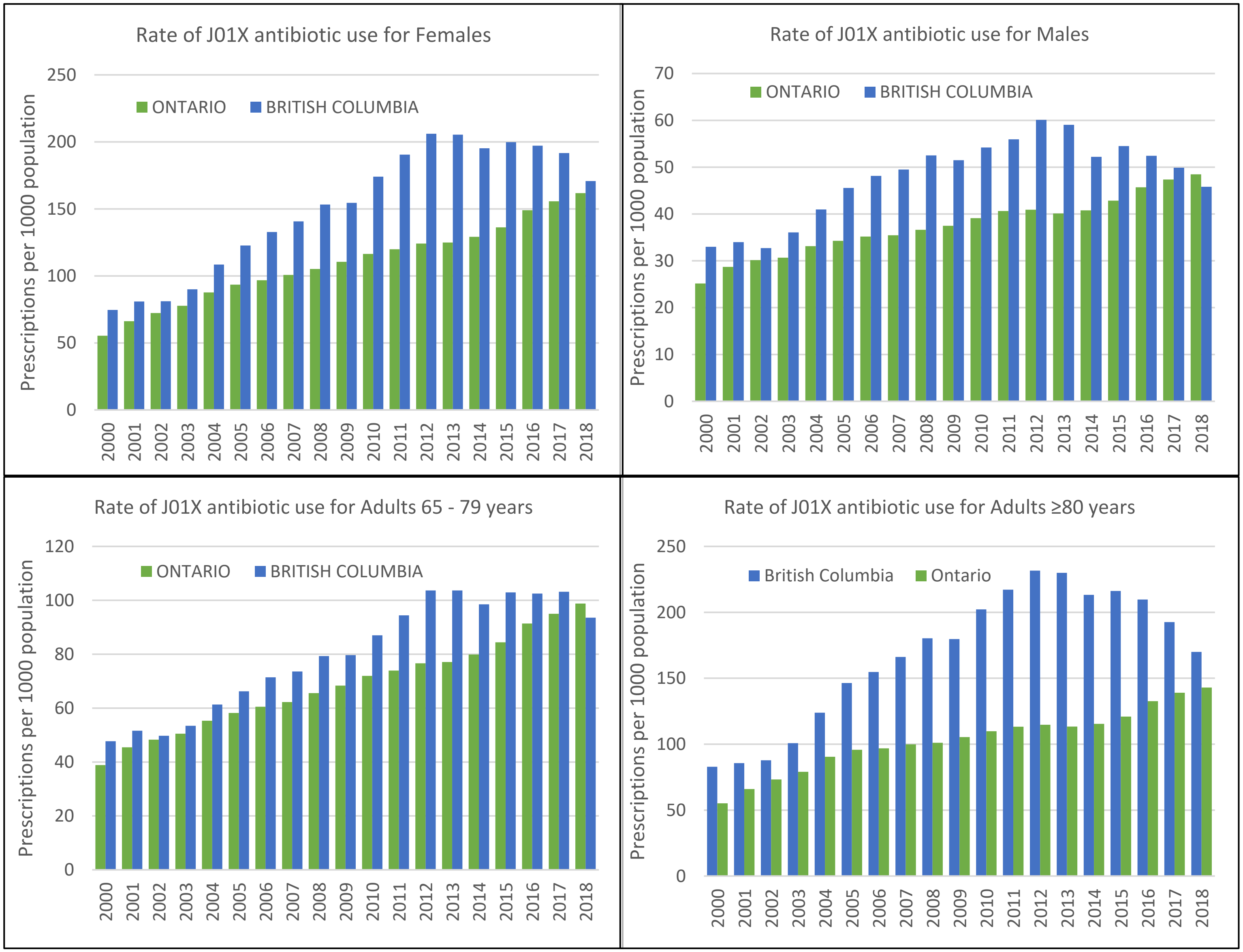

Supplement: Supplementary file 1 [file antibiotics-10-01484-s001.zip › antibiotics-1488353-supplementary.pdf]
